# *Micrococcus porci* sp. nov., Isolated from Feces of Black Pig (*Sus scrofa*)

**DOI:** 10.3390/life12111749

**Published:** 2022-10-31

**Authors:** Ai-Yun Lee, Chia-Hsuan Chen, Jong-Shian Liou, Yu-Chun Lin, Moriyuki Hamada, Yu-Ting Wang, Lin-Liang Peng, Shen-Chang Chang, Chih-Chieh Chen, Chuen-Fu Lin, Lina Huang, Chien-Hsun Huang

**Affiliations:** 1 Bioresource Collection and Research Center (BCRC), Food Industry Research and Development Institute, Hsinchu 30062, Taiwan; 2 Livestock Research Institute, Council of Agriculture, Executive Yuan, Tainan 71246, Taiwan; 3 Biological Resource Center, National Institute of Technology and Evaluation (NBRC), 2-5-8 Kazusakamatari, Kisarazu, Chiba 292-0818, Japan; 4 Division of Research and Analysis, Food and Drug Administration, Ministry of Health and Welfare, Taipei 11561, Taiwan; 5 Kaohsiung Animal Propagation Station, COA-LRI, Pingtung 91247, Taiwan; 6 Institute of Medical Science and Technology, National Sun Yat-sen University, Kaohsiung 80424, Taiwan; 7 Rapid Screening Research Center for Toxicology and Biomedicine, National Sun Yat-sen University, Kaohsiung 80424, Taiwan; 8 Department of Veterinary Medicine, College of Veterinary Medicine, National Pingtung University of Science and Technology, Pingtung 912301, Taiwan

**Keywords:** gut microbiota, *Micrococcus porci*, new taxa, polyphasic analysis

## Abstract

An aerobic bacterium, designated as strain KD337-16^T^, was isolated from the fecal samples of a black pig. It exhibited spherical, non-motile and non–spore-forming, Gram-positive cells. KD337-16^T^ was identified as a member of the genus *Micrococcus* through 16S rRNA gene sequencing, and its closest relatives were found to be *Micrococcus endophyticus* YIM 56238^T^ (99.5% similarity), *Micrococcus luteus* NCTC 2665^T^ (99.1%), *Micrococcus yunnanensis* YIM 65004^T^ (99.1%), *Micrococcus aloeverae* AE-6^T^ (99.1%), *Micrococcus antarcticus* T2^T^ (98.9%), and *Micrococcus flavus* LW4^T^ (98.7%). Phylogenomic trees were constructed, and strain KD337-16^T^ was found to form its own cluster as an independent lineage of *M. flavus* LW4^T^. Between KD337-16^T^ and its close relatives, the average nucleotide identity, average amino acid identity, and digital DNA–DNA hybridization were below the respective species delineation thresholds at 82.1–86.6%, 78.1–86.1%, and 24.4–34.9%. The major cellular fatty acids and polar lipids were anteiso-C_15:0_ and iso-C_15:0_, and DPG and PG, respectively. The predominant menaquinone was MK-8(H_2_). Taken together, the results indicate that strain KD337-16^T^ is a novel species of the genus *Micrococcus,* for which the name *Micrococcus* *porci* sp. nov. is proposed. The type strain is KD337-16^T^ (=BCRC 81318^T^ = NBRC 115578^T^).

## 1. Introduction

The genus *Micrococcus* was first described by Cohn [1], and this description was subsequently amended by Stackebrandt et al. [2] and Wieser et al. [3], with *Micrococcus luteus* as the type species. The genus *Micrococcus* belongs to the family *Micrococcaceae*, order *Micrococcales*, and phylum *Actinomycetota*. Nine species have been reported in this genus (https://lpsn.dsmz.de/genus/micrococcus, accessed on 28 October 2022): *Micrococcus* *aloeverae*, *Micrococcus* *antarcticus*, *Micrococcus* *cohnii*, *Micrococcus* *endophyticus*, *Micrococcus* *flavus*, *M. luteus*, *Micrococcus* *lylae*, *Micrococcus* *terreus*, and *Micrococcus* *yunnanensis*. Genotypically and phenotypically, the species *M. aloeverae*, *M. endophyticus*, *M. luteus*, *M. yunnanensis*, *M. antarcticus*, and *M. lylae* are closely related and together constitute the *M. luteus* group [4]. Previous studies have reported the isolation of *Micrococcus* strains from numerous habitats, including human skin [5], permanently cold samples [6], activated sludge [7], the inner tissues of plants [8,9,10], soil [11], dairy waste [12], air [13], and intestine of several fish species [14,15]. These strains have been described as emerging opportunistic pathogens [16,17].

Pig is not only an important protein source for the human diet, but has also become increasingly important as biomedical animal model of human beings. The pig growth trait has been found to correlate with the gut microbiota [18,19,20], and the *Prevotella copri* has demonstrated to regulate the fat accumulation through a causal study in pigs [21]. Culturomics is an approach that is based on diverse culture conditions and enables the maximal recovery of culturable microorganisms from biological samples [22,23]. The cultivation projects of swine gut microbiota have been completed in recent years [24,25,26]. However, a huge portion of intestinal bacteria have still not been cultured. For a better understanding of the physiological impact of the gut microbiome of the host, to isolate, identify and characterize of uncultured bacteria is necessary. During a study aimed at isolating novel bacterial species present in the pig intestine using culturomics, one isolated strain, KD337-16^T^ could not be assigned to any recognized species of the genus *Micrococcus* using matrix-assisted laser desorption ionization time-of-flight mass spectrometry (MALDI-TOF MS) analysis. Here, we report the phenotypic, chemotaxonomic and genotypic characterization of strain KD337-16^T^.

## 2. Materials and Methods

### 2.1. Isolation of Strain KD337-16^T^ and Culture Conditions

This study was approved by the Institutional Animal Care and Use Committee of Livestock Research Institute (permit no. LRI IACUC110-35). A fecal sample obtained from a KHAPS black pig (*Sus scrofa*) was collected from the Kaohsiung Animal Propagation Station in Pingtung County (approximate geographic coordinates: 22.63424° N 20.60237° E), Taiwan, in 2021. Wet-weight feces (1 g) were suspended in 10 mL of phosphate-buffered saline, and the suspension was subsequently homogenized. Serial dilutions were plated on blood agar that contained 5% sheep blood for 48 h of aerobic incubation at 37 °C. All of the isolates were subjected for strain dereplication using a MALDI Microflex LT mass spectrometer (Bruker Daltonics, Bremen, Germany), as previously described [27]. One strain, named KD337-16^T^, could not be identified confidently. Strain KD337-16^T^ and its phylogenetically closest reference species, including *M. endophyticus* BCRC 16908^T^, *M. luteus* BCRC 80739^T^, *M. yunnanensis* BCRC 80243^T^, *M. aloeverae* BCRC 80870^T^, and *M. flavus* BCRC 80069^T^, were routinely cultured on trypticase soy agar (TSA) at 37 °C for further taxonomic characterization, and the strains were then preserved in 10% glycerol at −80 °C.

### 2.2. DNA Extraction and Gene Sequence Comparison

A DNeasy kit (Qiagen, Valencia, CA, USA) was employed to extract and purify bacterial genomic DNA. The 16S rRNA gene and three housekeeping genes (*gyrB*, *recA*, and *rpoB*) were amplified and sequenced using a method reported elsewhere [4,28]. The EzBioCloud database (www.ezbiocloud.net/identify, accessed on 28 October 2022) was employed in subsequent BLAST analyses, and gene sequences were compared with those on NCBI GenBank (www.ncbi.nlm.nih.gov/BLAST/, accessed on 28 October 2022).

### 2.3. Phylogenetic Analysis

Clustal X (v. 2.1) software was employed for aligning sequences [29]. MEGA (v. 11) software was employed for phylogenetic tree reconstruction [30] based on sequences from the novel strain KD337-16^T^, its close relative strains, a roughly 1375 bp segment of the 16S rRNA gene, and nearly 1870 bp of the concatenated sequences of the three housekeeping genes (*recA*, *gyrB*, and *rpoB*). The neighbor-joining (NJ) [31], maximum-likelihood (ML) [32] and minimum-evolution (ME) [33] methods and the Kimura two-parameter model were used for tree reconstruction. Bootstrapping analysis with 1000 replicates was employed to determine how statistically reliable the trees were [34].

### 2.4. Genomic Analysis

A QIAamp PowerFecal Pro DNA Kit (Qiagen, Hilden, Germany) was employed to extract genomic DNA from the KD337-16^T^ strain. Subsequently, the SQK-LSK109 Ligation Sequencing Kit on a PromethION Flow Cell (R9.4.1) and Illumina NovaSeq 6000 in paired-end (2 × 151 bp) mode was used for Oxford Nanopore Technologies (ONT) sequencing. After the sequences had been decoded and refined, Flye version 2.8.3 was employed for assembly of the valid ONT sequences. The primary contigs were polished with Racon v1.4.22 and the Illumina read alignment results constructed using Minimap2 v2.17. The DDBJ Fast Annotation and Submission Tool was used to annotate the genome [35]. Methods described elsewhere [36,37,38] were employed to quantify the digital DNA–DNA hybridization (dDDH), the amino acid identity (AAI), and average nucleotide identity (ANI). The up-to-date bacterial core genes pipeline (http://leb.snu.ac.kr/ubcg2, accessed on 28 October 2022) [39] and EDGAR platform were utilized to construct phylogenomic trees [40], whereas the 3ggNOG 4.5 database and carbohydrate-active enzyme (CAZy) database were employed for functional assignment [41,42]. The OrthoVenn2 webserver was used for pangenome analysis [43]. Finally, AntiSMASH software (v. 6.0) was employed to predict putative biosynthetic gene clusters [44].

### 2.5. Phenotypic Characterization

For the phenotypic analysis, strain KD337-16^T^ was aerobically cultured on TSA at 37 °C for 48 h, unless otherwise stated. Cell morphology was observed under a phase-contrast microscope (Eclipse E600, Nikon, Tokyo, Japan). Gram staining was performed by using a Gram-staining kit (Difco, St. Louis, MA, USA) according to the manufacturer’s instructions. Growth at different temperatures (4, 15, 20, 28, 30, 37, 42 and 50 °C), NaCl concentrations (0–15% *w/v*, at 1% intervals) and pH levels (pH 4.0–12.0, at 1.0 pH unit intervals), tested in tryptic soy broth (TSB), were investigated through the standard methods [45]. Catalase activity was determined by the reaction of fresh cells toward 3% hydrogen peroxide (H_2_O_2_), and oxidase reaction was tested by using oxidase reagents (bioMerieux, Marcy-l’Étoile, France). The ability of the cells to utilize various sources of carbon and their enzyme activity were evaluated with commercial kits from API ZYM, API 20E, and Biolog GEN III MicroPlate system in accordance with the manufacturer’s instructions.

### 2.6. Chemotaxonomic Characterization

MALDI-TOF MS was performed for whole-cell protein analysis in accordance with a method described elsewhere [27]. Dendrogram clustering was constructed with the setting of 200 (distance measure: correlation; linkage: average; score oriented) using MALDI BioTyper software (v. 3.1; Bruker Daltonics, Billerica, MA, USA). Biomass for analysis of whole-cell fatty acids, polar lipids and isoprenoid quinone were obtained by culturing strain KD337-16^T^ in TSB for 2 days at 30 °C. A previously reported method and the Sherlock Microbial Identification System (MIDI) were used to analyze whole-cell fatty acids as fatty acid methyl esters [46]. Polar lipids were extracted from 100 mg freeze-dried cells using the method described by Minnikin et al. [47] and analyzed by TLC using chloroform/methanol/water (65:25:4, by vol.) in the first direction and chloroform/acetic acid/methanol/water (80:18:12:5, by vol.) in the second. Lipids were visualized by spraying the TLC plate with 10% molybdophosphoric acid. Anisaldehyde (sugar), Schiff’s reagent (glycol) and Dittmer–Lester reagent (phosphorous) were also used as specific spray reagents for polar lipids. The molecular species and concentrations of isoprenoid quinones were determined as described by Hamada et al. [48].

## 3. Results and Discussion

MALDI-TOF MS is a phenotype-based method that can be used for the rapid identification and dereplication of numerous isolates on the basis of specific proteomic profiles [49]. The MALDI-TOF MS spectra of type strain KD337-16^T^, shown in Supplementary Figure S1, could not be reliably identified, yielding a log score of <1.7. On the basis of pairwise sequence analysis of the 16S rRNA gene, strain KD337-16^T^ and its closest relatives, the type strains *M. endophyticus* YIM 56238^T^, *M. luteus* NCTC 2665^T^, *M. yunnanensis* YIM 65004^T^, *M. aloeverae* AE-6^T^, *M. antarcticus* T2^T^, and *M. flavus* LW4^T^, showed high similarity values of 99.5%, 99.1%, 99.1%, 99.1%, 98.9%, and 98.7%, respectively (Table 1).

**Figure 1:**
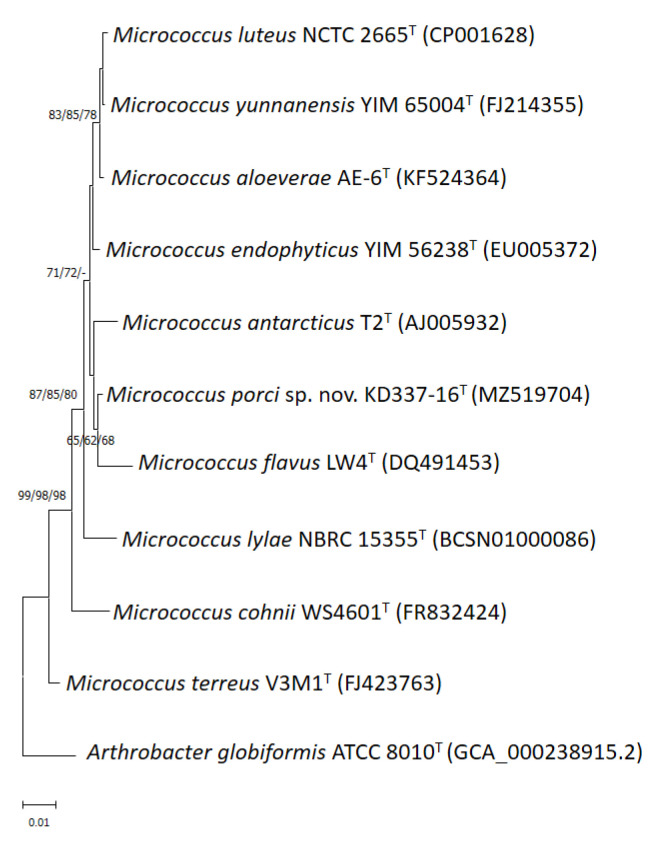
Phylogenetic tree based on 16S rRNA gene sequences showing the relationship of *M. porci* sp. nov. KD337-16^T^ with strains of closely related species. The tree was constructed by the neighbor-joining, minimum evolution and maximum-likelihood methods based on a comparison of approximately 1375 bp, and *Arthrobacter globiformis* ATCC 8010^T^ was used as the outgroup. The type strain of the *M. antarcticus* CGMCC 1.2372 and JCM 11467 is currently not available from a recognized culture collection. Bootstrap values (>60%) based on 1000 replicates are shown at branch nodes. Bar, 1% sequence divergence.

**Figure 2:**
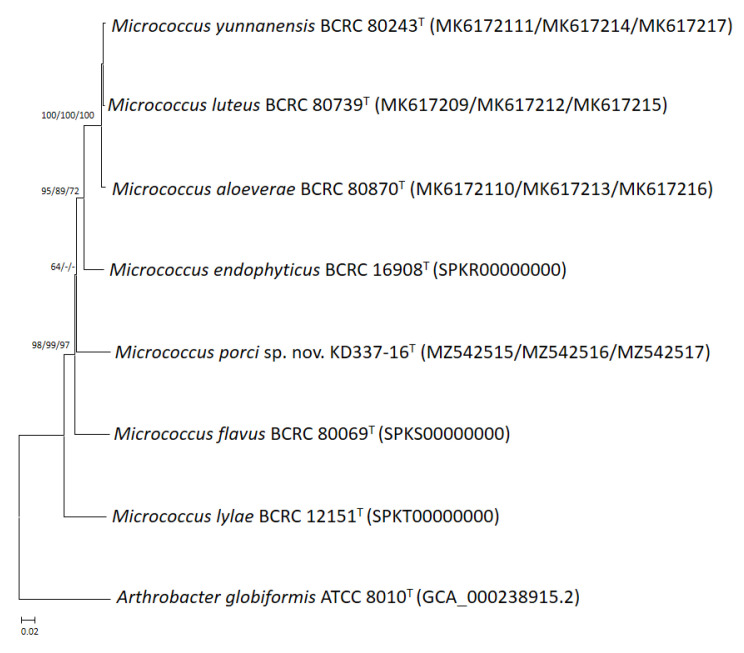
Phylogenetic tree based on the concatenated housekeeping gene sequences (*gyrB*, *recA*, and *rpoB*) showing the relationship of *M. porci* sp. nov. KD337-16^T^ with strains of closely related species. The tree was constructed by the neighbor-joining minimum evolution and maximum-likelihood methods based on a comparison of 1873 bp, and *A. globiformis* ATCC 8010^T^ was used as an outgroup. Bootstrap values based on 1000 replicates are shown at branch nodes. Bar, 2% sequence divergence.

Through phylogenetic analysis of 16S rRNA gene sequences, the strain was determined to be a member of the *M. luteus* group (Figure 1). Members of the *M. luteus* group can be discriminated with good resolution using protein-encoding genes such as *gyrB*, *recA* and *rpoB* [4]. Among these three, *gyrB* has the highest discriminatory power at the interspecific level. The degrees of similarity between KD337-16^T^ and other members of the *M. luteus* group were determined to be 90.1–91.6% (Table 1) on the basis of the concatenated sequences (*gyrB*, *recA*, and *rpoB*). When the NJ method was used for reconstruction, the resulting phylogenetic tree showed that strain KD337-16^T^ constituted its own cluster and that this cluster was clearly separate from that of its close relatives (Figure 2). Using the ME or ML method resulted in similar tree topology, which indicated that this strain may be a novel species.

**Figure 3:**
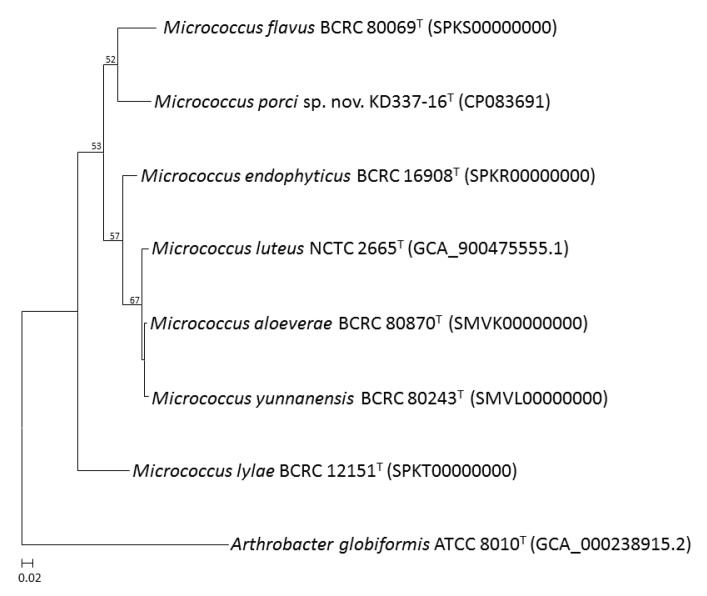
An UBCG tree based on 81 bacterial core genes of *M. porci* sp. nov. KD337-16^T^ and closely related species. Bootstrap values greater than 50% are shown at each node and *A. globiformis* ATCC 8010^T^ was used as an outgroup. Bar, 2% sequence divergence.

**Figure 4:**
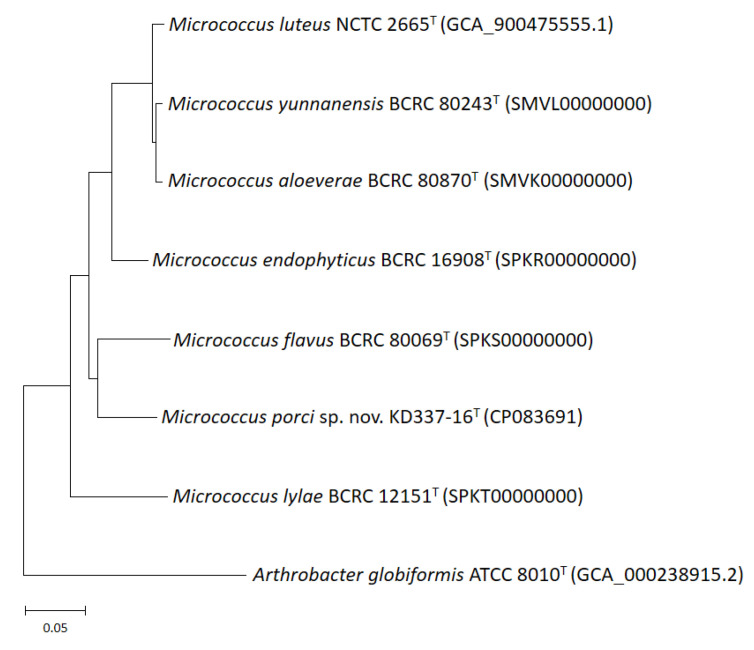
Phylogenomic tree of *M. porci* sp. nov. KD337-16^T^ with strains of closely related species. The tree was constructed by the maximum likelihood method the basis of a comparison of 368 core genes, and *A. globiformis* ATCC 8010^T^ was used as an outgroup. Bar, 5% sequence divergence.

Whole-genome sequencing is currently the most fruitful source of taxonomic information [50,51,52,53]. Comparative genomics, overall genome–related indices (e.g., AAI, ANI, and dDDH), and phylogenomic tree analyses may be critical approaches for estimating evolutionary distances among species and delineating prokaryotic taxa at the species and genus levels. The present study revealed that strain KD337-16^T^ had G+C content of 73.0% and a genome size of 2.64 Mb; the genome contained 2502 coding genes and 59 predicted RNA genes (Table 2). Between KD337-16^T^ and its close relations, the ANI, AAI, and dDDH varied from 82.1% to 86.6%, 78.1% to 86.1%, and 24.4% to 34.9%, respectively (Table 3), lower than 95–96%, 95%, and 70%, the respective generally accepted cutoffs for prokaryotic species. By contrast, the ANI, AAI, and dDDH values among the *M. aloeverae*, *M. luteus*, and *M. yunnanensis* type strains exceeded the species thresholds, indicating that the aforementioned strains are members of the same species [4]. Nevertheless, between *M. luteus* and *M. aloeverae* and between *M. luteus* and *M. yunnanensis*, the dDDH was 77.8% and 77.9%, respectively, below the threshold for subspecies delineation [54]. The phylogenomic trees were obtained on the basis of 81 and 368 core genes (Figure 3 and Figure 4) shared by the investigated strains and we also discovered an independent cluster formed solely by strain KD337-16^T^, thus confirming this strain’s status as a novel species. A total of 2313 genes from strain KD337-16^T^ were assigned to 21 functional categories (Supplementary Figure S2). The most common categories among these functional groups belonged to the [L] (recombination and repair; 220 genes) and [E] (amino acid transport and metabolism; 214 genes) clusters. Of identified CAZy families, the strain KD337-16^T^ contained 4 carbohydrate binding modules, 28 glycoside hydrolases, and 12 glycosyl transferases, respectively. In addition, 2012 orthologous protein clusters were discovered in KD337-16^T^. Of the shared clusters, 502 were common in the five *Micrococcus* species (Supplementary Figure S3), and 24 protein clusters were unique to the novel *Micrococcus porci* strain (Supplementary Table S1). KD337-16^T^ appears to produce putative secondary metabolite gene clusters, such as the beta lactone, terpene, and RiPP-like biosynthetic clusters.

Cells of strain KD337-16^T^ were coccoid shaped (approximately 0.5–1 µm) (Supplementary Figure S4), non-motile and non–spore-forming Gram-, catalase-, and oxidase-positive facultative anaerobic cells. Colonies were cream to yellow, slightly convex, smooth and circular. Table 4 lists the phenotypic characteristics that can be used to distinguish this novel strain from its close relatives. Cluster analysis of MALDI-TOF MS spectra in the 2000–12,000 *m*/*z* region of *Micrococcus* strains revealed the unambiguous grouping of five distinct clusters, each defined by known species and our novel taxon (Supplementary Figure S5). The fatty acid analysis revealed the major fatty acids (>10%) in strain KD337-16^T^ to be anteiso-C_15:0_ and iso-C_15:0_; furthermore, this novel strain can be differentiated by analyzing whether C_16:0_ is present and minor fatty acids such as iso-C_16:1_ H, anteiso-C_17:1_ω9c, and summed features 1 and 3 are absent (Table 5). The polar lipids of strain KD337-16^T^ were detected to be diphosphatidylglycerol, phosphatidylglycerol, phosphatidylinositol and unidentified glycolipid (Supplementary Figure S6). The predominant isoprenoid quinone of strain KD337-16^T^ was MK-8(H_2_); MK-7(H_2_) and MK-9(H_2_) were detected as minor components (85:14:1). These features were in agreement with those of the genus *Micrococcus* [55].

## 4. Conclusions

According to the results of polyphasic characterization, strain KD337-16^T^ is genetically and phenotypically discernible from other currently recognized *Micrococcus* species. Thus, it represents a novel taxon for which the name *Micrococcus porci* sp. nov. is proposed.

### Description of Micrococcus porci sp. nov.

*Micrococcus porci* (por’ci. L. gen. n. *porci* of a pig).

Cells are Gram-positive, aerobic, non-spore-forming, non-motile cocci with approximately 0.5–1 µm in diameter. Colonies are cream–yellow, slightly convex and smooth on TSA after 2 days at 37 °C under aerobic culture conditions. Growth occurs at 15–40 °C (not obviously at 15 °C) and pH 6–11, and tolerate 0–10% (*w/v*) NaCl. Cells are positive for catalase and oxidase. In API ZYM and API 20E system, positive reactions are obtained from the tests of alkaline phosphatase, esterase, esterase lipase, leucine arylamidase, valine arylamidase, cystine arylamidase, acid phosphatase, naphthol-AS-BI-phosphohydrolase, α-glucosidase, acetoin production and gelatinase, but not from lipase, trypsin, α-chymotrypsin, α-galactosidase, β-galactosidase, β-glucuronidase, β-glucosidase, *N*-acetyl-β-glucosaminidase, α-mannosidase, α-fucosidase, arginine dihydrolase, lysine decarboxylase, ornithine decar, boxylase, citrate utilization, urease, tryptophane deaminse and indole production. Biolog GEN III Microplate assays showed as positive for the utilization of dextrin, d-maltose, d-turanose, α-d-glucose, pectin, sodium butyrate, *p*-hydroxy-phenylacetic acid, l-lactic acid, α-hydroxy butyric acid, α-keto-butyric acid, potassium tellurite, d-fucose, l-fucose, acetoacetic acid and acetic acid; and negative for the utilization of d-trehalose, d-cellobiose, gentiobiose, sucrose, stachyose, d-raffinose, α-d-lactose, d-melibiose, β-methyl-d-glucoside, d-salicin, *N*-acetyl-β-d-mannosamine, *N*-acetyl galactosamine, *N*-acetyl neuraminic acid, d-mannose, d-fructose, 3-methyl glucose, myo-inositol, d-glucose-6-PO4, d-fructose-6-PO4, gelatin, l-alanine, l-arginine, l-aspartic acid, l-glutamic acid, l-histidine, l-pyroglutamic acid, d-gluconic acid, d-glucuronic acid, glucuronamide, mucic acid, quinic acid, d-saccharic acid, tetrazolium blue, methyl pyruvate, α-keto-glutaric acid, l-malic acid, bromo-succinic acid, Tween 40, propionic acid, sodium bromate, d-serine, troleandomycin, rifamycin SV, linomycin, tetrazolium violet, aztreonam, *N*-acetyl-d-glucosamine, d-aspartic acid, glycyl-l-proline, l-serine, d-lactic acid methyl ester, d-malic acid, γ-amino-butyric acid, β-hydroxy-d,l-butyric acid, d-galactose, l-rhamnose, d-galacturonic acid, l-galactonic acid lactone, fusidic acid, minocycline, guanidine HCl, niaproof 4, vancomycin, inosine, d-sorbitol, d-mannitol, d-arabitol, glycerol, citric acid and formic acid. The major cellular fatty acids and polar lipids are anteiso-C_15:0_ and iso-C_15:0_, and DPG and PG, respectively. The predominant menaquinone is MK-8(H_2_). The type strain KD337-16^T^ (=BCRC 81318^T^ = NBRC 115578^T^) was isolated from fecal samples of a black pig. The genome size of the type strain is 2.64 Mb and has a DNA G + C content of 73.0 mol%. The GenBank/EMBL/DDBJ accession numbers are MZ519704 (16S rRNA gene), MZ542515 (*gyrB* gene), MZ542516 (*recA* gene), MZ542517 (*rpoB* gene) and CP083691 (chromosome).

## Figures and Tables

**Table 1:** Sequence similarity of the *M. porci* sp. nov. KD337-16^T^ and its closely related species.

Species	Strain No.	Other Destination	Sequence Similarity (%) with Strain KD337-16^T^ *				
			16S rRNA	*gyrB*	*recA*	*rpoB*	MLSA ***
*M. endophyticus*	BCRC ** 16908^T^	YIM 56238^T^	99.5	86.9	96.3	92.9	91.6
*M. luteus*	BCRC 80739^T^	NCTC 2665^T^	99.1	86.7	94.4	91.8	90.6
*M. yunnanensis*	BCRC 80243^T^	YIM 65004^T^	99.1	87	94.4	91.7	90.5
*M. aloeverae*	BCRC 80870^T^	AE-6^T^	99.1	85.8	94.6	91.7	90.1
*M. antarcticus*	T2^T^	CGMCC 1.2372^T^	98.9	ND ****	ND	ND	ND
*M. flavus*	BCRC 80069^T^	LW4^T^	98.7	86.5	93.9	92.3	90.1

* T, type strain, ** BCRC, Bioresource Collection and Research Center at Food Industry Research and Development Institute, Taiwan. *** Calculated on the basis of the concatenated sequences of *gyrB*, *recA*, and *rpoB*. **** data not available.

**Table 2:** Genomic characteristics of *M. porci* sp. nov. KD337-16^T^ strain.

Attribute	KD337-16^T^
Accession no.	CP083691
Genome size (bp)	2,648,900
G+C content (mol%)	73.0
No. of contigs	1
N50 length (bp)	2,648,900
No. of CDS	2502
rRNAs	6
tRNAs	53
CRISPRS	0

**Table 3:** Average nucleotide identity (ANI), amino acid identity (AAI) and dDDH prediction values (%) between the strain KD337-16^T^ and its closely related species.

Species	Strain	Accession No	1	2	3	4	5	6
1. *M. porci* sp. nov.	KD337-16^T^	CP083691	100.0	34.0	24.4	34.9	34.4	24.6
2. *M. endophyticus*	BCRC 16908^T^	SPKR00000000	86.6 ^a^/83.2 ^b^	100.0	43.3	44.2	44.0	32.7
3. *M. luteus*	NCTC 2665^T^	GCA_900475555.1	82.1/78.1	90.9/89.9	100.0	77.8	77.9	23.2
4. *M. yunnanensis*	BCRC 80243^T^	SMVL00000000	86.3/86.1	91.0/91.7	97.1/97.6	100.0	85.0	30.9
5. *M. aloeverae*	BCRC 80870^T^	SMVK00000000	86.6/86.0	90.6/91.4	97.5/97.8	98.1/98.6	100.0	31.0
6. *M. flavus*	BCRC 80069^T^	SPKS00000000	82.2/80.2	86.4/83.6	80.6/77.4	84.9/84.4	85.4/84.2	100.0

The numbers show on the upper right are the dDDH values (%), and the ANI ^a^ and AAI ^b^ values (%) are shown on the lower left.

**Table 4:** Differential characteristics of *M. porci* sp. nov. KD337-16^T^ and the phylogenetically closest related species of the genus *Micrococcus*. Strains: 1, KD337-16^T^; 2, *M. endophyticus* BCRC 16908^T^; 3, *M. luteus* BCRC 11034^T^; 4, *M. flavus* BCRC 80069^T^. All four strains were tested in parallel in this study for reactions of API ZYM, API 20E, and BIOLOG GENIII. +, Positive; −, Negative. ND, data not available. DPG, diphosphatidylglycerol; PG, phosphatidylglycerol; PI, phosphatidylinositol; PC, phosphatidylcholine; PL, unidentified phospholipids; GL, unidentified glycolipids.

Characteristic	1	2	3	4
Temperature range for growth (°C)	15–40	15–37	20–40	26–34
pH range for growth	6–11	6–9	5–10	5–9
API ZYM results:				
Esterase	+	−	+	+
Valine arylamidase	+	−	+	+
Cystine arylamidase	+	−	−	−
Trypsin	−	−	+	−
α-Chymotrypsin	−	−	+	+
Acid phosphatase	+	−	+	−
Naphthol-AS-BI-phosphohydrolase	+	+	−	+
API 20E results:				
Arginine dihydrolase	−	+	−	−
Urease	−	−	+	−
Gelatinase	+	−	−	−
BIOLOG GEN III results:				
4% NaCl	+	−	−	−
8% NaCl	+	−	−	−
1% Sodium lactate	+	−	+	−
Dextrin	+	−	+	−
d-Maltose	+	−	−	−
d-Turanose	+	−	−	−
d-Melibiose	−	−	+	−
α-d-Glucose	+	−	−	−
d-Fructose	−	−	+	−
3-methyl glucose	−	−	+	−
d-Glucose-6-PO4	−	+	+	−
d-Fructose-6-PO4	−	−	+	−
Pectin	+	−	+	−
d-Gluconic acid	−	−	+	−
d-Glucuronic acid	−	−	+	−
Glucuronamide	−	−	+	−
Mucic acid	−	−	+	−
Sodium butyrate	+	+	+	−
Sodium bromate	−	+	−	−
Linomycin	−	+	−	−
Aztreonam	−	+	+	−
*p*-Hydroxy-phenylacetic acid	+	−	−	−
l-Lactic acid	+	−	−	−
α-Hydroxy butyric acid	+	−	−	−
α-keto-Butyric acid	+	−	+	−
Potassium tellurite	+	+	+	−
d-Galactose	+	−	+	−
d-Fucose	+	−	+	−
l-Rhamnose	−	−	+	−
d-Galacturonic acid	−	−	+	−
l-Galactonic acid lactone	−	−	+	−
Polar lipids *				
	DPG, PG, PI, GL	DPG, PG, PI, PL	DPG, PG, PI, PL, GL	ND
Respiratory quinones *				
	MK-8(H_2_), MK-7(H_2_), MK-9(H_2_)	MK-8(H_2_), MK-7(H_2_)	MK-8, MK-8(H_2_)	MK-8(H_2_), MK-7(H_2_)

* Data for the type strains were taken from Liu et al. [7], Chen et al. [8] and Rieser et al. [13].

**Table 5:** Cellular fatty acid compositions (%) of *M. porci* sp. nov. KD337-16^T^ and the phylogenetically closest related species of the genus *Micrococcus*. Scheme 1. KD337-16^T^; 2, *M. endophyticus* YIM 56238^T^; 3, *M. luteus* DSM 20030^T^; 4, *M. flavus* CGMCC 1.5361^T^ [8]. Values are percentages of total fatty acids. Fatty acids present at >10% are indicated in bold. TR, Trace amount (<1.0%). –, not detected.

Fatty Acid	1	2	3	4
Saturated				
C_14:0_	1.85	TR	1.05	TR
C_16:0_	4.12	TR	TR	-
Unsaturated				
C_14:1_*ω*5*c*	-	-	TR	TR
C_15:1_*ω*6*c*	-	TR	-	TR
Branched-chain fatty acids				
iso-C_13:0_	4.70	TR	TR	3.98
iso-C_14:0_	2.31	2.59	1.88	3.98
iso-C_15:0_	**35.04**	**30.95**	**14.67**	**23.35**
iso-C_16:0_	2.96	1.42	5.22	0.34
iso-C_16:1_ H	-	TR	1.03	1.13
iso-C_17:0_	TR	TR	TR	-
anteiso-C_13:0_	1.10	TR	TR	2.78
anteiso-C_15:0_	**45.01**	**53.75**	**63.16**	**41.92**
anteiso-C_17:0_	1.66	1.36	6.03	-
anteiso-C_17:1_*ω*9*c*	-	TR	1.46	1.27
Summed feature *				
1	-	4.19	TR	**17.56**
3	-	TR	2.53	1.31

* Summed feature 1 comprises iso-C_15:1_ H and/or C_13:0_ 3-OH; summed feature 3 comprises C_16:1_ω7c and/or C_6:1_ω6c.

## Data Availability

The sequencing data has been uploaded to GenBank (MZ519704, MZ542515~MZ542517, and CP083691).

## Supplementary Materials

The following supporting information can be downloaded at: https://www.mdpi.com/article/10.3390/life12111749/s1, Figure S1: Representative spectra from *M. porci* sp. nov. KD337-16^T^ type strain. Figure S2: Results of an eggNOG functional category analysis of 2313 genes. Figure S3: Venn diagram showing the number of core, shared and unique genes (orthologous clusters) for each genome. Figure S4: Electron microscopic photograph of strain KD337-16^T^ after aerobic cultivation on TSA plate at 37 °C for 1 day. Figure S5: Dendrogram showing the clustering of the *Micrococcus* strains based on MALDI-TOF MS analysis. Figure S6: Two-dimensional thin-layer chromatograms of polar lipids from strain KD337-16^T^. Table S1: Genomic characteristics of *M. porci* sp. nov. KD337-16^T^ strain.
