# Supplementary material for: Micrococcus porci sp. nov., Isolated from Feces of Black Pig (Sus scrofa)
Source: Life (Basel). 2022 Oct 31;12(11):1749. doi: 10.3390/life12111749 (PMC9697426; doi:10.3390/life12111749)
Supplement: Supplementary file 1 [file life-12-01749-s001.zip › Supplementary Figures_20221019.pdf]

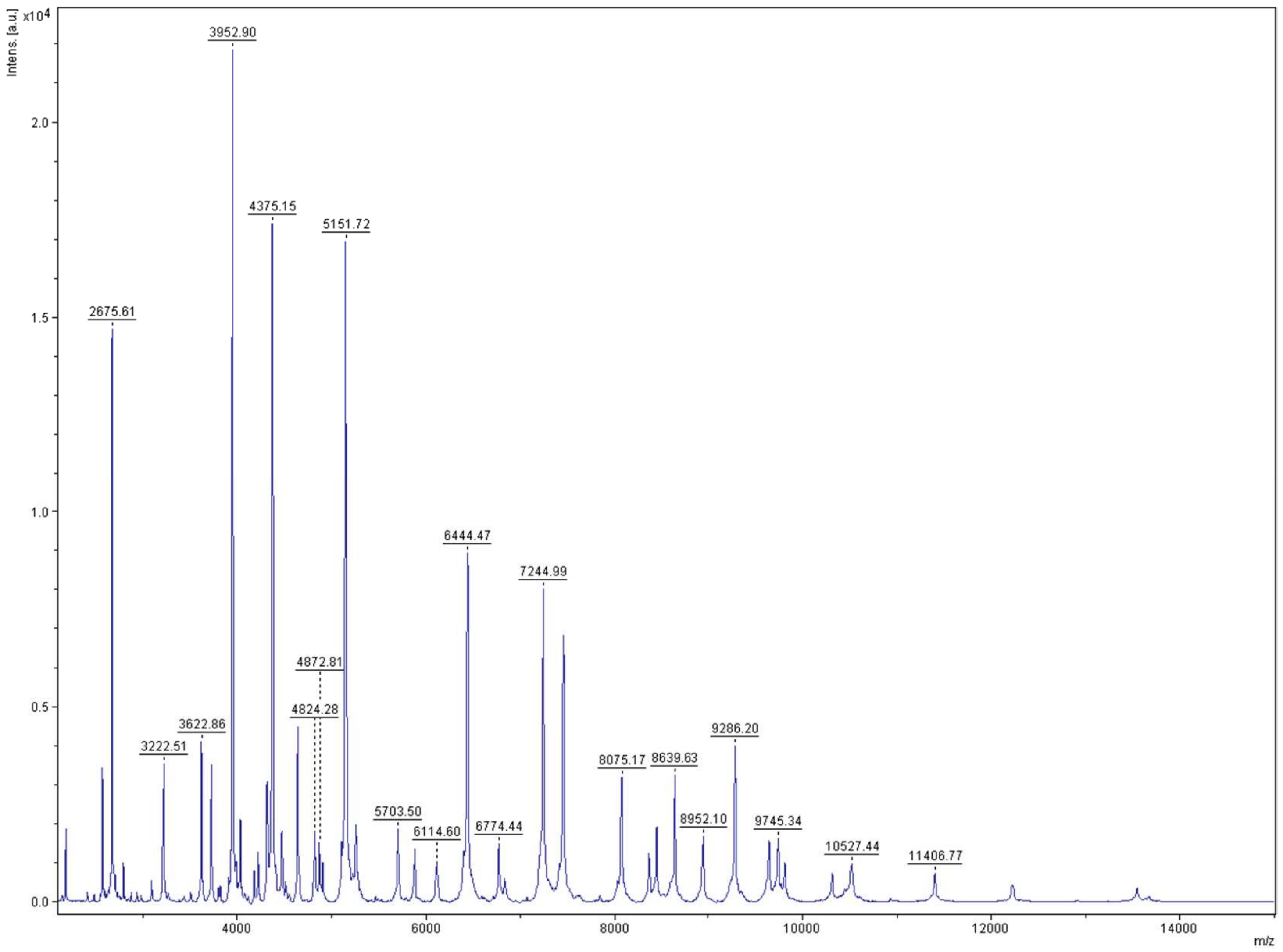

Supplementary Figure S1. Representative spectra from *M. porci* sp. nov. KD337-16^{T} type strain.

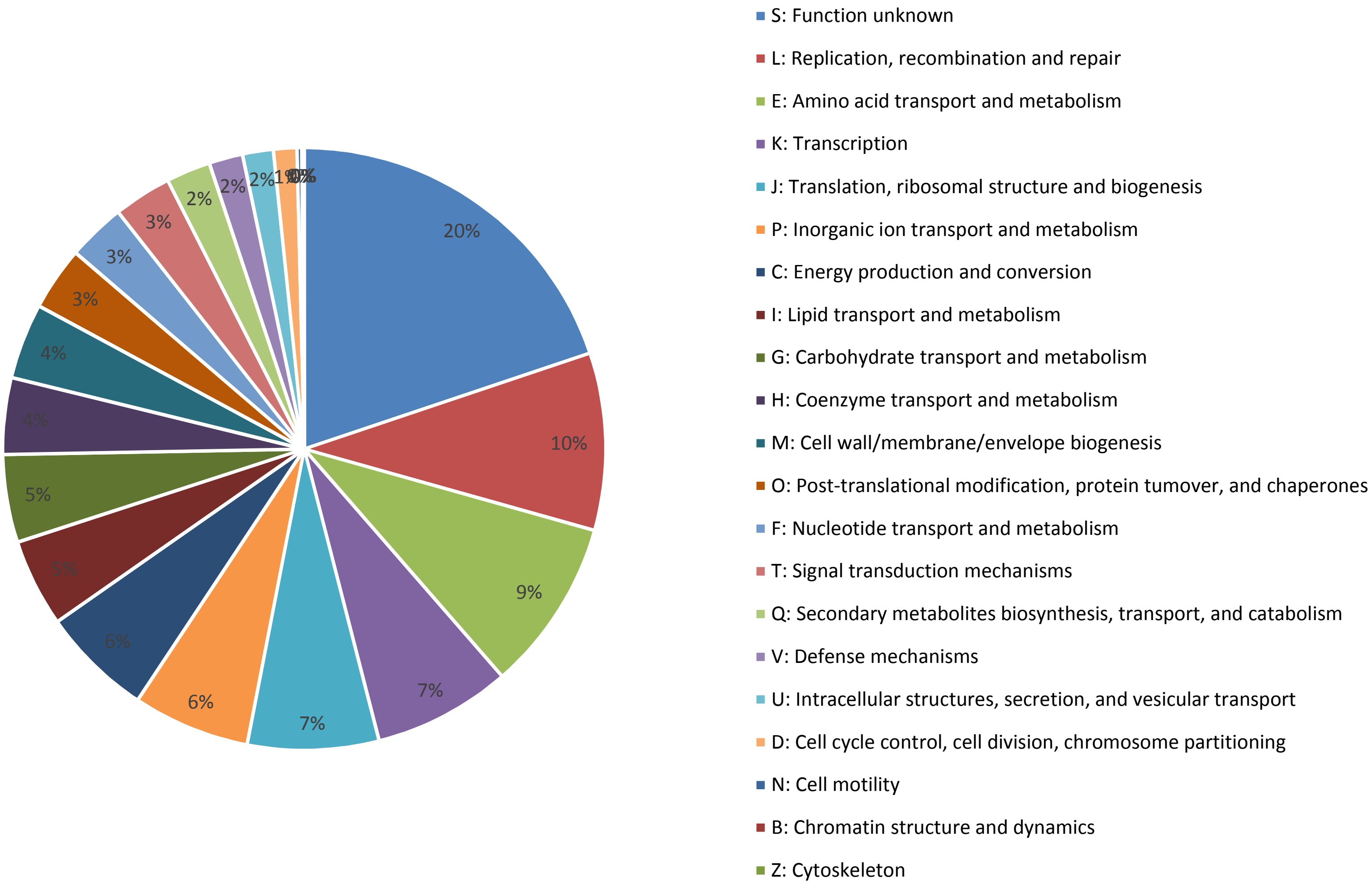

Supplementary Figure S2. Results of an eggNOG functional category analysis of 2,313 genes. The major three parts are COG category L (Replication, recombination and repair) and E (Amino acid transport and metabolism), respectively.

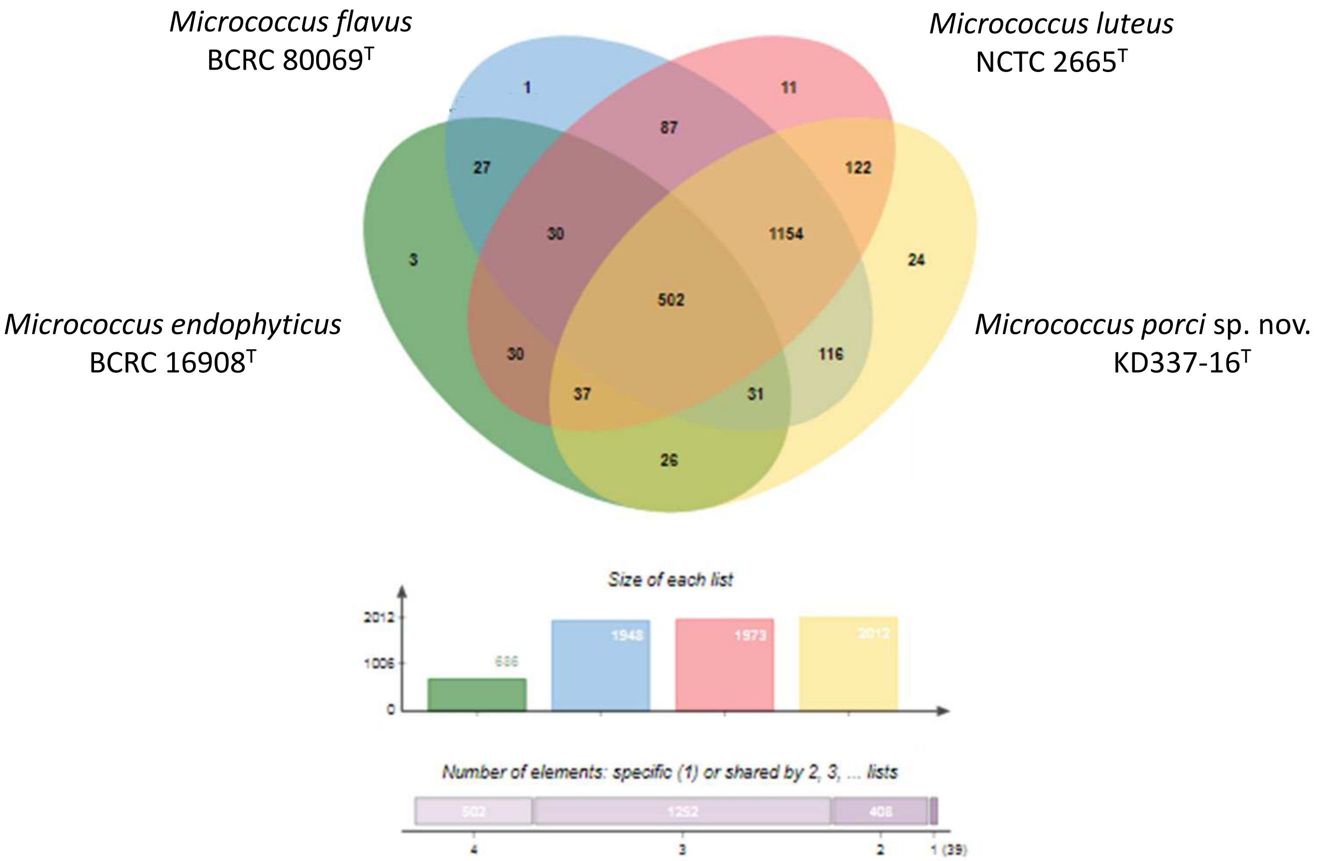

Supplementary Figure S3. Venn diagram showing the number of core, shared and unique genes (orthologous clusters) for each genome.

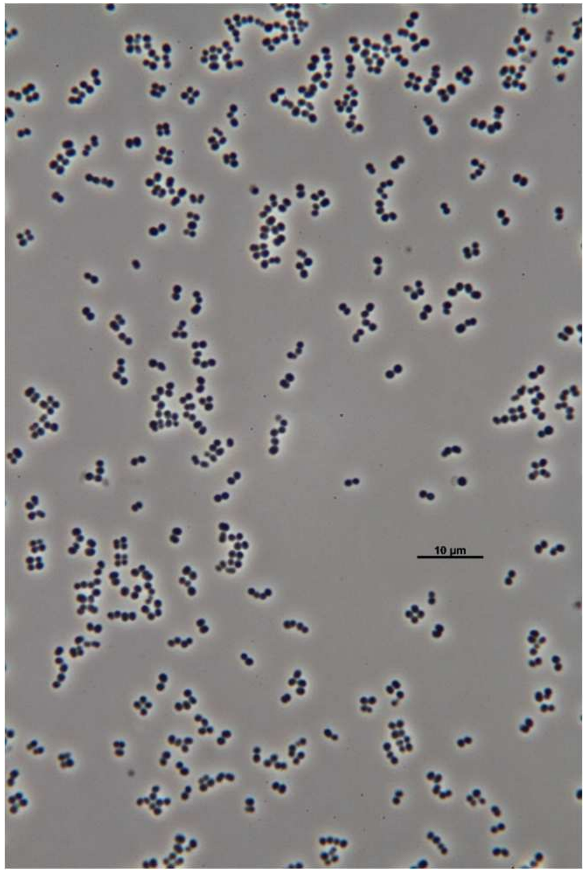

Supplementary Figure S4. Electron microscopic photograph of strain KD337-16^T after aerobic cultivation on TSA plate at 37 $^\circ$C for 1 day.

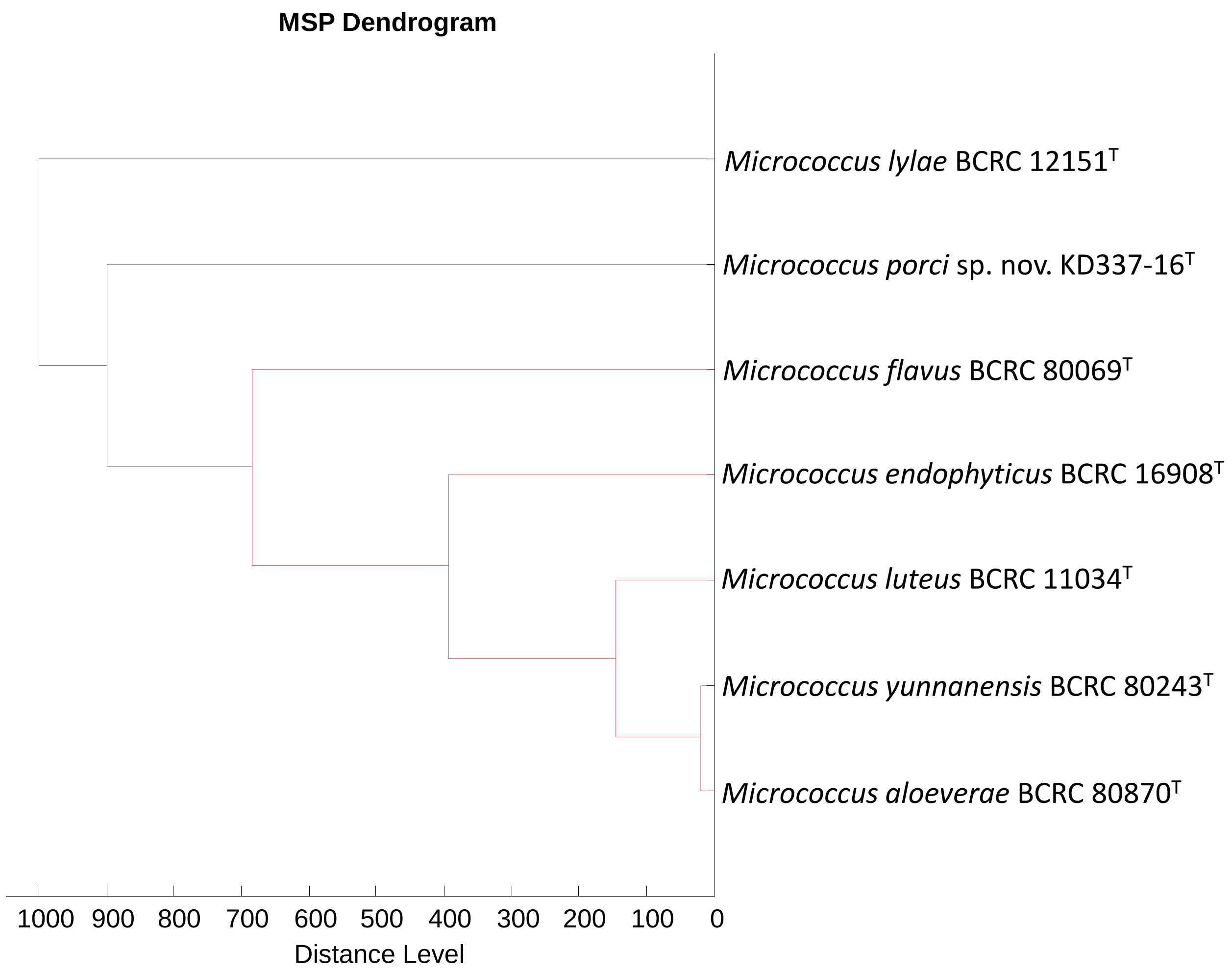

Supplementary Figure S5. Dendrogram showing the clustering of the *Micrococcus* strains based on MALDI-TOF MS analysis.

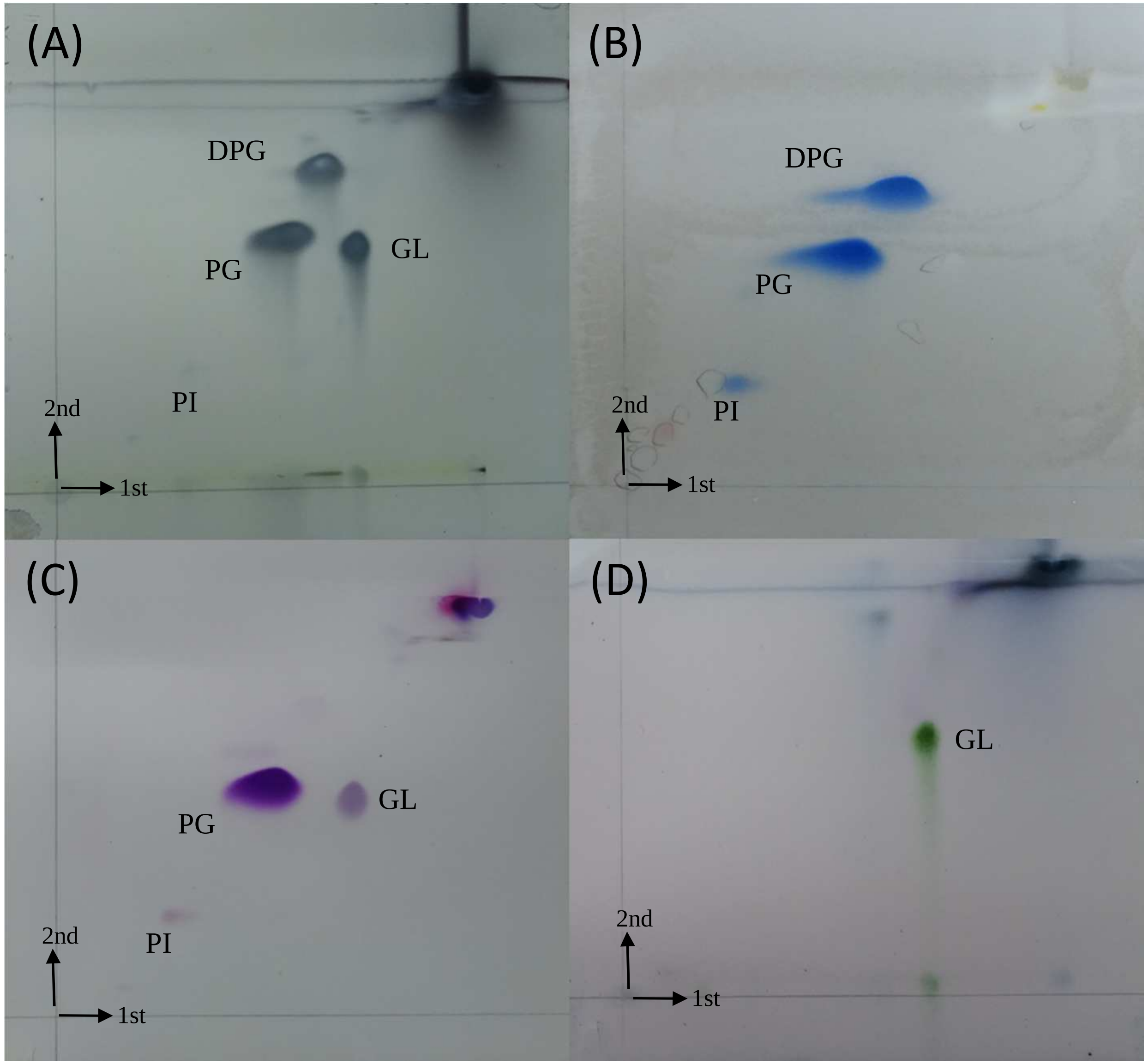

Supplementary Figure S6**.** Two-dimensional thin-layer chromatograms of polar lipids from strain KD337-16^{T}. The spray reagents; 10% molybdophosphoric acid (A), Dittmer-Lester reagent (B), periodic acid-Schiff's reagent (C) and anisaldehyde (D). Chloroform-methanol-water (65:25:4) by volume was used in the first direction and chloroform-acetic acid-methanol-water (80:18:12:5) by volume in the second direction. DPG, diphosphatidylglycerol; PG, phosphatidylglycerol; PI, phosphatidylinositol; GL, unidentified glycolipid.
