# Supplementary material for: Micrococcus porci sp. nov., Isolated from Feces of Black Pig (Sus scrofa)
Source: Life (Basel). 2022 Oct 31;12(11):1749. doi: 10.3390/life12111749 (PMC9697426; doi:10.3390/life12111749)
Supplement: Supplementary file 1 [file life-12-01749-s001.zip › Supplementary Table S1_20221019.pdf]

Supplementary Table S1. The novel protein clusters of *Micrococcus porci* sp. nov. KD337-16^{T}

Cluster name	Protein number	Swiss-Prot Hit	GO annotation
cluster38	4	P69927	P:response to antibiotic; IEA:UniProtKB-KW KD33716|LOCUS_06850;KD33716
cluster40	4	N/A	N/A
cluster531	3	N/A	N/A
cluster1785	2	N/A	N/A
cluster1786	2	N/A	N/A
cluster1787	2	N/A	N/A
cluster1788	2	O07434	GO:0046688; P:response to copper ion; IDA:MTBBASE
cluster1789	2	Q6AHI5	):0015103; F:inorganic anion transmembrane transporter activity; IEA:UniProtKB-UniRule
cluster1790	2	N/A	N/A
cluster1791	2	Q5SKN9	GO:0046872; F:metal ion binding; IEA:UniProtKB-KW
cluster1792	2	P16898	GO:0046677; P:response to antibiotic; IEA:UniProtKB-KW
cluster1793	2	N/A	N/A
cluster1794	2	Q4WZ69	GO:0035837; P:ergot alkaloid biosynthetic process
cluster1795	2	N/A	N/A
cluster1796	2	N/A	N/A
cluster1797	2	N/A	N/A
cluster1798	2	N/A	N/A
cluster1799	2	N/A	N/A
cluster1800	2	Q829X3	GO:0009432; P:SOS response; IEA:UniProtKB-UniRule
cluster1801	2	N/A	N/A
cluster1802	2	N/A	N/A
cluster1803	2	O00087	GO:0019464; P:glycine decarboxylation via glycine cleavage system; ISO:PomBase
cluster1804	2	N/A	N/A
cluster1805	2	N/A	N/A
